# Population genomics of *Zea* species identifies selection signatures during maize domestication and adaptation

**DOI:** 10.1186/s12870-022-03427-w

**Published:** 2022-02-18

**Authors:** Gen Xu, Xuan Zhang, Wenkang Chen, Renyu Zhang, Zhi Li, Weiwei Wen, Marilyn L. Warburton, Jiansheng Li, Huihui Li, Xiaohong Yang

**Affiliations:** 1 State Key Laboratory of Plant Physiology and Biochemistry, National Maize Improvement Center of China, MOA Key Laboratory of Maize Biology, China Agricultural University, Beijing, 100193 China; 2 Joint International Research Laboratory of Crop Molecular Breeding, China Agricultural University, Beijing, 100193 China; 3 Key Laboratory of Horticultural Plant Biology (MOE), College of Horticulture and Forestry Sciences, Huazhong Agricultural University, Wuhan, 430070 China; 4 United States of Department of Agriculture, Agricultural Research Service, Corn Host Plant Resistance Research Unit, Box 9555, Mississippi, MS 39762 USA; 5 Institute of Crop Sciences, Chinese Academy of Agricultural Sciences, Beijing, 100081 China

**Keywords:** *Zea*, Evolutionary relationship, Domestication, Adaptation, Genome-wide association study, Flowering time

## Abstract

**Background:**

Maize (*Zea mays* L. ssp. *mays*) was domesticated from teosinte (*Zea mays ssp. parviglumis*) about 9000 years ago in southwestern Mexico and adapted to a range of environments worldwide. Researchers have depicted the maize domestication and adaptation processes over the past two decades, but efforts have been limited either in sample size or genetic diversity. To better understand these processes, we conducted a genome-wide survey of 982 maize inbred lines and 190 teosinte accessions using over 40,000 single-nucleotide polymorphism markers.

**Results:**

Population structure, principal component analysis, and phylogenetic trees all confirmed the evolutionary relationship between maize and teosinte, and determined the evolutionary lineage of all species within teosinte. Shared haplotype analysis showed similar levels of ancestral alleles from *Zea mays ssp. parviglumis* and *Zea mays ssp. mexicana* in maize. Scans for selection signatures identified 394 domestication sweeps by comparing wild and cultivated maize and 360 adaptation sweeps by comparing tropical and temperate maize. Permutation tests revealed that the public association signals for flowering time were highly enriched in the domestication and adaptation sweeps. Genome-wide association study identified 125 loci significantly associated with flowering-time traits, ten of which identified candidate genes that have undergone selection during maize adaptation.

**Conclusions:**

In this study, we characterized the history of maize domestication and adaptation at the population genomic level and identified hundreds of domestication and adaptation sweeps. This study extends the molecular mechanism of maize domestication and adaptation, and provides resources for basic research and genetic improvement in maize.

**Supplementary Information:**

The online version contains supplementary material available at 10.1186/s12870-022-03427-w.

## Background

Maize (*Zea mays* L. ssp. *mays*) is the most widely planted crop species for food, feed, and industrial materials [1]. Maize, along with its wild relatives, also serves as an excellent model organism for understanding the genetic and functional mechanisms of plant domestication and adaptation. Maize and teosinte make up the genus *Zea*, which consists of five species distributed from northern Mexico through Central America [2–4]. The five species are *Zea nicaraguensis* (hereafter *nicaraguensis*), *Zea luxurians* (hereafter *luxurians*), *Zea diploperennis* (hereafter *diploperennis*), *Zea perennis* (hereafter *perennis*), and *Zea mays*. Of these, *diploperennis* and *perennis* are diploid and tetraploid perennial teosinte, respectively, whereas the others are diploid annual species. The annual species *Zea mays* consists of four subspecies, including the domesticated maize, the lowland adapted *Zea mays* ssp. *parviglumis* (hereafter *parviglumis*), the highland adapted *Zea mays* ssp. *mexicana* (hereafter *mexicana*), and the mid-altitude adapted *Zea mays* ssp. *huehuetenangensis* (hereafter *huehuetenangensis*). A refined understanding of the genetic relationship within the genus *Zea* can help elucidate the trajectories of maize domestication and adaptation.

Previously published genetic and archaeological data clearly reveal that maize was domesticated from *parviglumis* in a single domestication event in southern Mexico ~ 9000 years ago [5–7]. During this period, maize underwent dramatic phenotypic changes in both morphological and physiological characteristics [8–10]. The genetic basis of the morphological differences between maize and teosinte has been intensely investigated by quantitative trait locus (QTL) mapping using maize-teosinte populations [11–15]. However, only a limited number of domestication QTLs have been mapped to the underlying genes, including *teosinte branched1* (*tb1*) controlling branching [16–18], *teosinte glume architecture1* (*tga1*) controlling the formation of the stony fruit case [19, 20], and *grassy tillers1* (*gt1*) affecting prolificacy [21]. In addition to the cloning of single genes, population genetics comparisons of maize and teosinte have revealed evidence for positive selection in hundreds of genes during maize domestication [3, 22].

After its domestication, maize began to spread from southern Mexico into North and South America, where it adapted to these diverse environmental conditions [4, 5]. One of the most important events in this adaptation process was the divergence between tropical and temperate lines around 3400–6700 years ago [23]. Various environmental differences between temperate and tropical regions, such as temperature and day length, shaped maize diversity and facilitated its movement, and the footprints of this adaptation process were recorded in its genome. As with the study of the domestication process, genome-wide-level genotypic datasets provide an excellent resource for characterizing the genetic basis of adaptation. Adaptation studies involving these datasets linked to many aspects of the maize phenotypes and its metabolic pathways have identified a large number of selected loci, which reveal the complex genetic architecture of adaptation [23–25].

Flowering time is a key component in the adaptation of maize to local conditions as it moved to higher latitudes post-domestication. Five *cis*-variants in four genes, including a miniature transposon (MITE) located ~ 70 kb upstream of *ZmRap2.7* [26], a CACTA-like transposon in the *ZmCCT10* promoter [27], a Harbinger-like transposon located ~ 57 kb upstream of *ZmCCT9* [28], and SNP-1245 and InDel-2339 in the promoter of *ZCN8* [29], have been identified that contribute to phenotypes that allowed the pre-Columbian spread of maize throughout the Americas. The map of these interacting genes suggests that the SNP-1245A allele of *ZCN8* may have been the first to be selected, whereas the other four early-flowering alleles made specific contributions to northward expansion in North America [29]. These results suggest that the adaptation of maize was a complex process, involving numerous genetic loci that were selected at different evolutionary times for local adaptation [23].

During the past two decades, researchers have depicted the history of maize domestication and adaptation using genetic information from cultivated maize and its wild relatives [3, 5, 7, 22–24], but efforts have been limited either in sample size or geographic range. Here, a collection of 982 maize inbred lines representing global tropical, subtropical, and temperate germplasm and 190 teosinte accessions from Mexico and Central America were genotyped using the Illumina MaizeSNP50 BeadChip. We used this resource to determine the evolutionary relationship of the genus *Zea*, and to identify the loci that have undergone selection during maize domestication and adaptation. Subsequently, we performed co-localization analysis of selective sweeps with known selected genes, and associated genes for adaptation traits identified via genome-wide association studies (GWASs). We found that parts of the selected loci were associated with domestication and adaptation traits. This study will provide insights into maize evolutionary history, and the genetic resource should facilitate future maize breeding.

## Results

### Genetic structure within the genus *Zea*

**Fig. 1:**
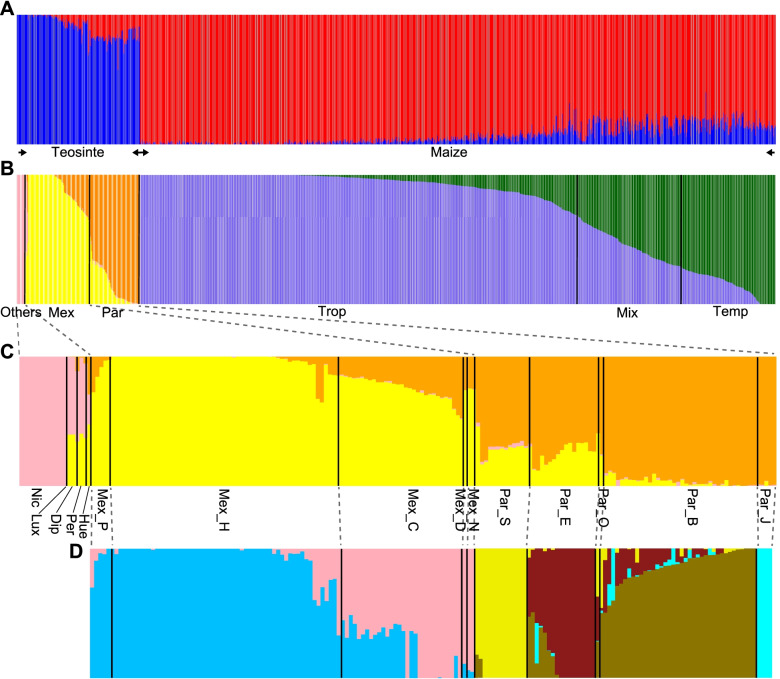
Population structure determined by ADMIXTURE for 982 maize lines and 190 teosinte accessions. **A** All 1172 materials were unambiguously divided into maize and teosinte groups. **B** The 982 maize and 190 teosinte entries were further subdivided into two and three groups, respectively. **C** The enlarged distribution plot of membership probabilities of the 190 teosinte entries in each group. **D** The 96 *mexicana* and 75 *parviglumis* accessions were clustered into two and four clusters, respectively. In *parviglumis*, one Par_O race is inferred as a separate, mixed group, as it has equal membership probabilities in three groups. In *mexicana*, two Mex_N and one Mex_D races clustered with the Mex_C race. The corresponding race names are shared with those in **C**. In (**A-D**), each individual is represented by a vertical bar, and colored segments indicate the estimated membership probabilities for that individual in the corresponding clusters (Data S1). Trop, tropical maize; Temp, temperate maize; Mix, maize lines with membership probabilities in both groups of < 0.70; Nic, *nicaraguensis*; Lux, *luxurians*; Dip, *diploperennis*; Per, *perennis*; Hue, *huehuetenangsis*; Mex, *mexicana*; Par, *parviglumis*. The *parviglumis* races: Par_S, Southern Guerrero; Par_E, Eastern Balsas; Par_O, Oaxaca; Par_B, Central Balsas; Par_J, Jalisco. The *mexicana* races: Mex_P, Puebla; Mex_C, Central Plateau; Mex_D, Durango; Mex_H, Chalco; Mex_N, Nobogame

Using 42,204 high-quality single-nucleotide polymorphisms (SNPs), all 1172 materials (982 maize inbred lines and 190 teosinte accessions) were unambiguously assigned to the maize or the teosinte clusters through population structure analysis (Fig. 1A; Data S1). Membership probabilities of each teosinte individual in the maize cluster (0 < *P* < 0.5) reflected the common ancestry between some teosinte accessions and maize. Maize inbred lines were further divided into tropical/subtropical (hereafter tropical; 669 lines) and temperate (157 lines) subgroups (Fig. 1B; Data S1), consistent with the historical separation of these two subgroups [5, 30]. A substantial mixed group (156 lines) also shows the effect of more recent breeding efforts to expand diversity within each breeding pool by bringing in germplasm from the other. Twelve teosinte accessions from *nicaraguensis* and one accession from *luxurians* clustered into a single subgroup (Fig. 1B, C; Data S1), suggesting the possibility of genetic similarity between these two subspecies. Accessions from *mexicana* and *parviglumis* clustered independently, and each formed a unique subgroup with 96 and 75 accessions, respectively (Fig. 1B, C; Data S1). The *diploperennis*, *perennis*, and *huehuetenangsis* accessions clustered into a mixed subgroup, and the membership probabilities of *diploperennis* and *perennis* in *mexicana* and *nicaraguensis* subgroups were similar (Fig. 1C; Data S1). Subsequent differentiation of *mexicana* and *parviglumis* revealed two major subgroups including two *mexicana* clusters and four *parviglumis* clusters, in agreement with races classified by geographical distribution (Fig. 1D; Fig. S1; Data S1).

In addition to the population structure analysis, we also carried out a principal component analysis (PCA) using the same SNP data set, and found that the PCA results strongly supported the classification of species, subspecies, and races based on the population structure analysis of the genus *Zea* (Fig. S2). Whereas, the PCA plots show that the extreme points in maize represent B73 and Mo17, and that the spread of the maize points is distorted and over-stretched. This phenomenon might be caused by SNP ascertainment bias, especially from the Syngenta SNPs. To evaluate the effect of ascertainment bias caused by Syngenta SNPs, we re-analyzed population structure and principal component using 30,974 non-Syngenta SNPs. The results from ADMIXTURE show the correlation of membership probabilities calculated by all SNPs and non-Syngenta SNPs are pretty high (*R*^2^ > 0.99) for each assigned group (Fig. S3A, B). In addition, the PCA plots show similar distribution of maize (Fig. S3C, D). Furthermore, we calculated the polymorphic information content (PIC) for each SNP, and found that the genetic diversity was quite similar between the results calculated from two different datasets (Fig. S3E). Taken together, these results suggest that the ascertainment bias caused by Syngenta SNPs did not affect the global estimation of genetic relationship and genetic diversity in the genus *Zea* although it indeed affected the genetic distance of maize inbred lines.

**Fig. 2:**
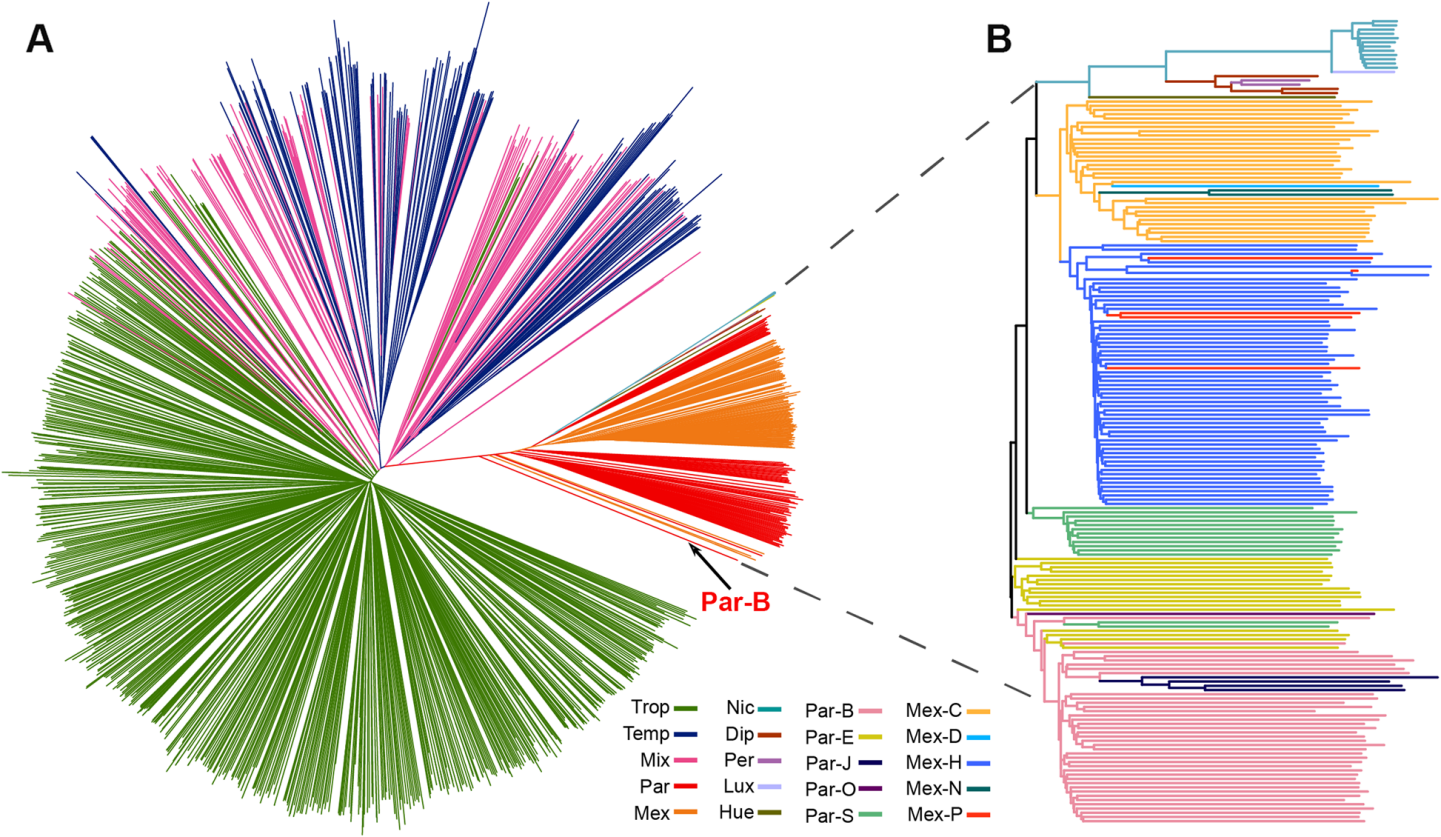
Neighbor-joining phylogenetic tree of maize and teosinte lines rooted with one *nicaraguensis* accession. **A** The genetic relationship of all maize plus teosinte. The *parviglumis* accessions from the Central Balsas race (Par_B) were closest to maize although four mexicana accessions from three races (Mex_H, Mex_P, Mex_C) were mixed among Par_B. **B** The genetic relationship of all teosinte accessions and all races in *parviglumis* and *mexicana*. All materials are marked according to the inferred clusters from the ADMIXTURE analysis. Trop, tropical maize; Temp, temperate maize; Mix, maize lines with membership probabilities in both tropical and temperate groups of < 0.70; Nic, *nicaraguensis*; Lux, *luxurians*; Dip, *diploperennis*; Per, *perennis*; Hue, *huehuetenangsis*; Mex, *mexicana*; Par, *parviglumis*. The *parviglumis* races: Par_S, Southern Guerrero; Par_E, Eastern Balsas; Par_O, Oaxaca; Par_B, Central Balsas; Par_J, Jalisco. The *mexicana* races: Mex_P, Puebla; Mex_C, Central Plateau; Mex_D, Durango; Mex_H, Chalco; Mex_N, Nobogame

To identify the primary sources of maize genetic diversity, we constructed a neighbor-joining phylogenetic tree that included all entries in this study (Fig. 2). In the phylogenetic tree, the accession in the *luxurians* group was closest to *nicaraguensis* (chosen as the root of the tree), followed in order by *diploperennis* and *perennis*, *huehuetenangsis*, *mexicana*, *parviglumis*, and, finally, maize. These groupings reflect the evolutionary lineage of all *Zea* species and subspecies. The monophyletic clade including all maize lines (Fig. 2A) strongly supports a single domestication event in maize. The *parviglumis* accessions from the Central Balsas race were closest to maize (Fig. 2), favoring the Balsas River valley as the center of maize domestication [5, 6, 31, 32]. In addition, the groups formed by the *mexicana* and *parviglumis* accessions seemed to be interconnected in a manner consistent with their geographical overlap (Fig. 2B; Fig. S1). Collectively, the evolutionary relationship of all *Zea* species and subspecies inferred by three methods is fully consistent with the current taxonomy of the genus *Zea*.

### Shared and unique haplotypes in maize and teosinte

Because of their proximity to maize, further analyses were focused on *mexicana* and *parviglumis* teosinte, as compared with tropical and temperate maize. These comparisons allowed the determination of genetic variation acquired by maize from teosinte during the domestication period, as compared to variation partitioned during its adaptation from tropical to temperate environments. High pairwise *F*_ST_ among these four subgroups (0.10 < *F*_ST_ < 0.21) indicated high population differentiation (Table S1). Furthermore, high pairwise *F*_ST_ between teosinte and maize and relatively small *F*_ST_ between tropical and temperate maize reflect maize domestication and adaptation history. Whereas, we found the haplotype richness in *parviglumis* was similar with that in tropical maize (Table 1). To exclude the biased estimation of haplotypes caused by sample size, we randomly selected 75 samples in each group with 100 bootstraps except *parviglumis* that had the smallest sample size. As expected, the window-based haplotype number in teosinte was much greater than modern maize, with the order following as *parviglumis* > *mexicana* > tropical maize > temperate maize (Fig. S4). These findings indicate that the genetic diversity in maize, especially temperate maize, was dramatically reduced during maize domestication and adaptation.

**Table 1:** Summary statistics of window-based haplotypes for all lines, groups, and subgroups in this study

Groups	All entries	Maize			Teosinte		
		Overall	Tropical	Temperate	Overall	*mexicana*	*parviglumis*
Sample size	1172	982	671	148	190	96	75
Number of haplotypes	98,177	85,863	81,928	66,359	86,770	74,032	78,605
Number of haplotypes per locus	5.74	5.02	4.79	3.88	5.07	4.33	4.59
Haplotype richness	6.73	5.84	5.55	4.41	5.9	4.98	5.31
Group-specific haplotypes	23,721	11,407	4421	640	12,314	2536	4346
Group-specific haplotypes/line	20.24	11.62	6.59	4.32	64.81	26.42	57.95
Group-specific haplotype (%)	0.24	13.29	5.40	0.96	14.19	3.43	5.53

**Fig. 3:**
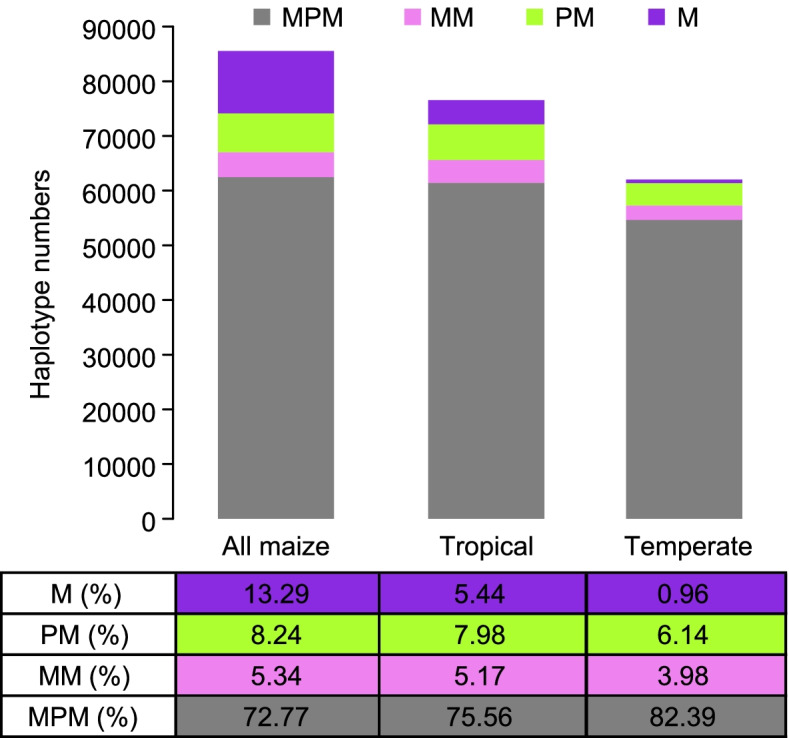
Shared ancestral haplotypes found in different maize groups, including all maize, tropical and temperate maize. The bottom table shows the percentage of each component. MPM, the haplotypes shared among *mexicana*, *parviglumis*, and maize; MM, the haplotypes shared only between *mexicana* and maize; PM, the haplotypes shared only between *parviglumis* and maize; M, the private haplotypes from maize

Many group-specific haplotypes were also observed in the four subgroups, *parviglumis*, *mexicana*, tropical and temperate maize (Fig. 3; Table 1). The presence of relatively fewer maize-specific haplotypes suggests that most of the diversity present in the domesticated maize gene pool is contributed by teosinte, and is not due to de novo haplotype creation since domestication. Both tropical and temperate maize had a great proportion of haplotypes in common with *parviglumis* and *mexicana* (Fig. 3), suggesting that both *parviglumis* and *mexicana* contributed to ancestral alleles into domesticated maize. Whereas, the contribution of *parviglumis* to maize during domestication may be overestimated because of the rapid expansion of the initial maize progenitor population.

### Footprints of selection in the genome

**Fig. 4:**
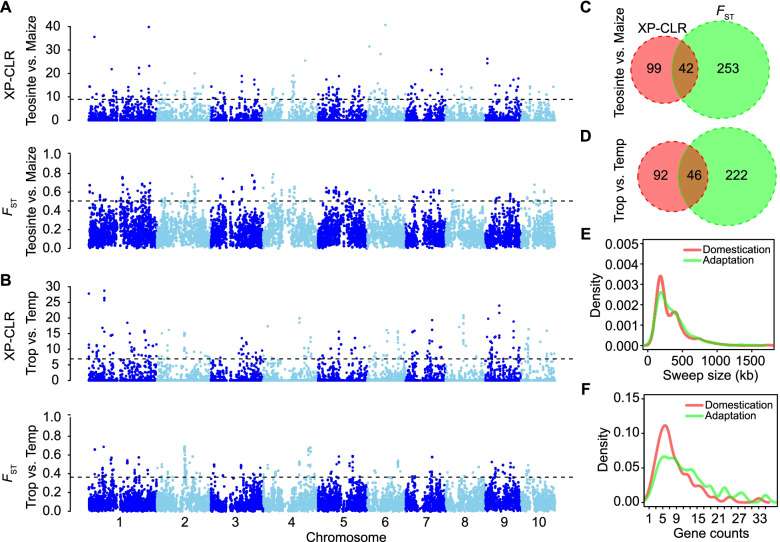
Genome-wide scan for regions that have undergone selection during maize domestication and adaptation. **A**, **B** Whole genome screening for regions that have undergone selection during maize domestication (comparing teosinte and maize) (**A**) and adaptation (comparing tropical and temperate maize) (**B**). The horizontal dashed lines indicate the genome-wide significance threshold of selection signals (top 0.5%). **C**, **D** Venn diagrams of the common and specific selective sweeps with domestication (**C**) and adaptation (**D**) features identified by two methods. **E**, **F** Distributions of sweep size (**E**) and gene counts within selective sweeps (**F**) in domestication and adaptation scans. XP-CLR, cross-population composite likelihood ratio; *F*_ST_, fixation index. Temp, temperate maize; Trop, tropical maize

The domestication of maize from its wild progenitor resulted in extreme morphological changes in plant and ear architecture, followed by further changes as a result of selection during crop adaptation [8, 33]. To determine if these changes can be detected as footprints of selection in the maize genome, two between-population comparisons, the calculation of *F*_ST_, and a cross-population composite likelihood ratio (XP-CLR) approach, were implemented for sliding windows between teosinte and maize, and between tropical and temperate maize (Fig. 4; Table 2; Data S2). Based on the top 0.5% of XP-CLR and *F*_ST_ values, we identified 141 and 295 regions, respectively, associated with domestication, with 42 regions identified in common by both methods (Fig. 4C; Table 2). We similarly identified 138 and 268 regions, respectively, for adaptation, with 46 regions identified by both methods (Fig. 4D; Table 2). The small portion of overlapping sweeps (~ 30%) between different methods may be due to the different aspects the two methods focus on. *F*_ST_ is based on single marker analysis with large variance of its measurements, while XP-CLR is a model-based extension of *F*_ST_ to multiple-loci analysis using linkage disequilibrium (LD) in the reference population to weight SNPs and then to reduce the high ratio of false positives [34]. Collectively, we identified 394 regions with domestication features and 360 regions with adaptation features, covering 5.7% (131 Mb) and 5.5% (127 Mb) of the genome, respectively (Table 2). For domestication, the size of these selection footprint regions ranged from 100 kb to 1.7 Mb, with a mean size of 333 kb, harboring 2218 genes; fewer selection footprint regions with a similar average size (352 kb) were detected during the adaptation process (Fig. 4E, F; Table 2). In addition, 69 of the domestication-related selective sweeps showed evidence of selection during adaptation, indicating that a subset of around 17% of the domestication loci may have also contributed to adaptation related phenotypes (Data S2).

**Table 2:** Summary of selective sweeps identified during maize domestication and adaptation

Selection Feature	Method	Selective sweeps			Gene number
		Number	Average length (kb)	Range (kb)	
Domestication	*F*_ST_	295	386	110–1730	1759
	XP-LCR	141	166	100–420	585
	Total^a^	394	333	100–1730	2218
Adaptation	*F*_ST_	268	412	120–1530	2181
	XP-LCR	138	171	100–340	622
	Total^a^	360	352	100–1530	2665

^a^shows the characteristics of the selective sweeps identified jointly by two methods

**Fig. 5:**
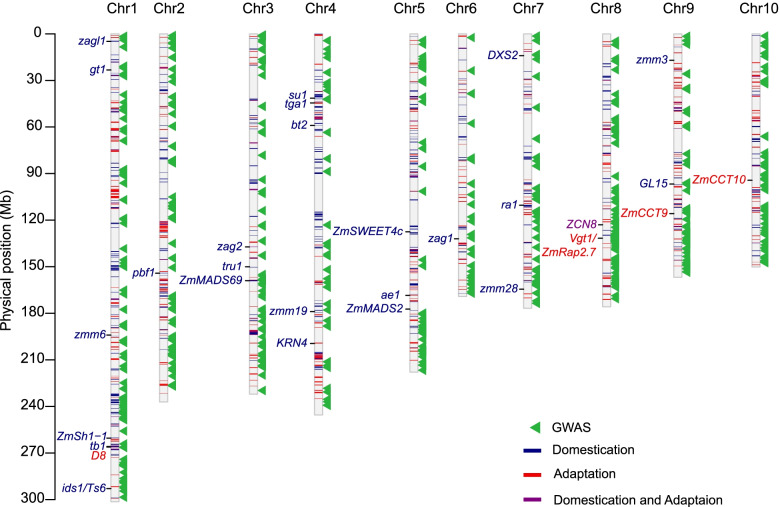
Overview of regions experiencing selection and their co-localization with the loci for flowering-time traits. The physical position of GWAS signals (green triangles) from previously published studies [27, 39, 40] are shown for flowering-time traits. The blue and red lines within each chromosome indicate the location of domestication and adaptation sweeps found in this study, and the purple lines indicate the common selection sweeps detected in both the domestication and adaptation processes. Known domestication genes and flowering-time genes are also shown in their mapped locations

To test if genetic variation within selected regions contributed to phenotypic changes during maize domestication and adaptation, we collected 29 previously reported genes with evidence of selection during domestication and adaptation (Table S2) and performed a co-localization analysis (Fig. 5; Data S2). Of the 29 genes, nine genes fell within the selective sweeps detected in our study, and eight genes which were previously reported to be domestication-related genes were physically located within the domestication-related selective sweeps identified here, i.e. *tb1* [18, 35], *pbf1* [36], and *zagl1* [37, 38]. The finding that we didn’t identify all the 29 known selected genes may be a consequence of the low marker density or different germplasms. Taken together, our results provide evidence that some selective sweeps identified here are associated with domestication traits, although the causative genes in most selective sweeps remain unknown.

### Selection footprint regions associated with adaptation traits

To mine more loci or genes under selection during the adaptation process, we are using flowering-time traits as a representative for adaptation traits, since flowering time plays a key role in the process of adaptation that allowed maize to spread so widely [27–29]. We performed an additional co-localization analysis between selection sweeps and genomic regions associated with flowering-time traits as identified by genome-wide association studies (GWASs) [27, 39, 40]. A total of 32 domestication (8.1%) and 39 adaptation (10.8%) sweeps were co-located with GWAS signals for flowering time (Fig. 5; Data S2). Then we carried out a 1000-permutation test using the randomly sampled genomic regions with the same number and size as the selective sweeps compared to these public GWAS hits for flowering time [27, 39, 40]. The results revealed that the GWAS signals for flowering time were highly enriched in the domestication and adaption sweeps (Permutation test, *P* < 0.001) (Fig. S5). Notably, three reported flowering-time genes, *ZmMADS69* [41], *PhyB1* [42, 43], and *zmm3* [37, 44], were detected within the GWAS signals as well as the selective sweeps. These results suggest that the genes underlying these co-localized regions for flowering-time traits might have undergone selection during maize domestication and adaptation.

**Fig. 6:**
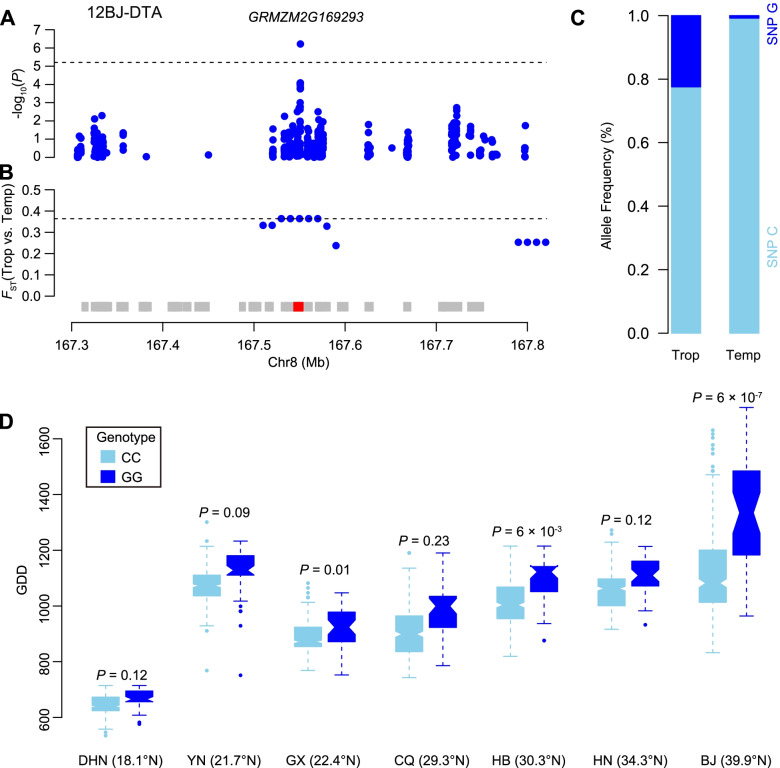
The gene *GRMZM2G169293* associated with flowering time was selected during maize adaptation. **A** Associations between SNPs at the *GRMZM2G169293* locus and flowering time. The dashed black horizontal line indicates the Bonferroni-adjusted significance threshold (*P* = 6.05 × 10^− 6^). **B** The *F*_ST_ values for selection during adaptation across the *GRMZM2G169293* locus. The horizontal dashed line indicates the genome-wide significance threshold of selection signals (top 0.5%). Red and gray boxes indicate the gene models of *GRMZM2G169293* and other genes. **C** The allele frequency of the leading SNP (SNP S8_167550959) at the *GRMZM2G169293* locus in tropical and temperate maize inbred lines. **D** Association tests of SNP S8_167550959 with flowering time in seven locations at different latitudes. Days to anthesis (DTA) were converted to growing degree days (GDD) to account for the effect of temperature differences among environments. DHN, Hainan; YN, Yunnan; GX, Guangxi; CQ, Chongqing; HB, Hubei; HN, Henan; BJ, Beijing; all in China

In addition to characterization of selected regions potentially related to flowering time, we compared our selected regions to a marker-trait association mapping that was done for four flowering-time traits using a set of 508 maize inbred lines with ~ 1.25 million SNPs [45]. At a *P*-value ≤6.05 × 10^− 6^ (1/165,202), a total of 10, 6, 11, and 4 loci were significantly associated with days to anthesis (DTA), days to silking (DTS), anthesis photoperiod response (APR), and silking photoperiod response (SPR), respectively (Data S3) when using best linear unbiased prediction (BLUP) values. Comparison of our selective sweeps to this GWAS on flowering-time traits using the set of 508 inbred lines grown at seven locations at diverse latitudes was also instructive; that GWAS identified 188 additional SNPs that resolved to 106 loci, and ten co-located with adaptation-related selective sweeps (Table 3; Data S3). The function of these ten candidate genes for flowering time that underwent selection during maize adaptation were annotated as transcription factors, flavonol synthase, MYB DNA-binding domain superfamily protein, *etc* (Table 3). Of these loci, association and adaptation-related selective signals were both noted at the gene *GRMZM2G169293* (Fig. 6A, B), which encodes a ceramide and inositol phosphotransferase. We found that 77% of tropical inbred lines carried the C allele at the SNP (S8_167550959) that showed the most significant association at the *GRMZM2G169293* locus, and that the percentage of lines with the C allele increased to 99% among temperate inbred lines (Fig. 6C). These contrasting frequency distributions suggest that the C allele of SNP S8_167550959 might be associated with distinct patterns of geographic dispersal. Interestingly, SNP S8_167550959 exhibited significant association with flowering time only at high latitudes, and the effects increased with latitude (except within Yunnan, China; Fig. 6D). Although the function of *GRMZM2G169293* affecting flowering time need more solid evidence, i.e., overexpression or mutant analysis, these findings suggested the characterization of genes responsible for adaptation from tropical to temperate regions.

**Table 3:** Summary of maize adaptation loci that were significantly associated with flowering-time traits

Candidate Gene^a^	Lead trait^b^	Lead SNP^c^	Position^d^	Allele^e^	MAF^f^	*P*-value	Other trait^g^	Annotation
*GRMZM2G382569*	DTS_BJ	S1_62146730	Chr1_62146730	G/T	0.05	6.0 × 10^−6^		Flavonol synthase/flavanone 3-hydroxylase
*GRMZM2G180847*	DTA_GX	S2_194077681	Chr2_194077681	T/C	0.13	1.5 × 10^−6^	DTS_GX	Probable transcription factor PosF21
*GRMZM2G151044*	DTA_YN	S4_180919965	Chr4_180919965	C/T	0.10	3.7 × 10^−6^		Trehalose-6-phosphate phosphatase4
*GRMZM2G111537*	DTA_HB	S5_31012027	Chr5_31012027	A/C	0.48	5.1 × 10^−7^	APR_BLUP/DTS_HB	Adenine nucleotide alpha hydrolases-like superfamily protein
*GRMZM5G812923*	DTA_DHN	S5_81991055	Chr5_81991055	G/A	0.09	1.3 × 10^−6^	DTS_DHN	Protein SCO1 homolog 1 mitochondrial
*GRMZM2G147867*	DTA_YN	S6_147732326	Chr6_147732326	G/A	0.07	1.1 × 10^−6^		NAC domain-containing protein 35
*GRMZM2G435034*	DTA_BJ	S8_156956875	Chr8_156956875	C/T	0.10	1.1 × 10^−6^		MACPF domain-containing protein
*GRMZM2G169293*	DTA_BJ	S8_167550959	Chr8_167550959	C/G	0.10	6.0 × 10^−7^		Ceramide inositol phosphotransferase 1
*GRMZM2G166780*	DTA_GX	S9_139587213	Chr9_139587213	C/T	0.15	4.5 × 10^−6^		THO complex subunit 4B
*GRMZM2G425427*	DTA_BJ	S10_85333950	Chr10_85333950	C/G	0.08	6.6 × 10^−9^		Putative MYB DNA-binding domain superfamily protein

^a^A plausible biological candidate gene at the locus or the nearest annotated gene to the lead associated SNP

^b^The trait with the most significant association signals among the tested traits. The trait is named by the abbreviation of flowering-time traits and locations for field trails. APR, anthesis photoperiod response; SPR, silking photoperiod response; DTA, days to anthesis; DTS, days to silking. DHN, Hainan; YN, Yunnan; GX, Guangxi; CQ, Chongqing; HB, Hubei; HN, Henan; BJ, Beijing; BLUP, best linear unbiased prediction

^c^The SNP with the most significant association signals for the lead trait at a given locus, where the major allele is underlined

^d^Chromosome and physical position for SNPs according to B73 RefGenV3 reference genome

^e^The major allele for each SNP is underlined

^f^MAF, minor allele frequency

^g^The remaining associated traits at a given locus

## Discussion

The germplasm analyzed here is comprised of an ecologically diverse collection of species including domesticated maize from tropical and temperate regions, and its close wild relatives. These taxa provide an excellent genetic resource to address multiple questions about speciation and evolution, structural and functional genomics, and utilization of teosinte germplasm in maize breeding. Cultivated maize has experienced a long period of artificial selection for desirable traits such as high yield (e.g., large seeds), nutrient richness (e.g., high levels of starch, oil, carotenoids, etc.), and ease of harvest [8–10, 15, 46]. This productivity-directed selection process generally results in the loss of genetic diversity in maize and an increased vulnerability to biotic and abiotic stresses [9].

Comparison of polymorphism data between maize landraces and teosinte in previous studies report a substantial loss (17%) of diversity during the domestication bottleneck [47]. Following further and more intense artificial selection, modern maize lost even more (18.6%) genetic diversity compared to teosinte [47]. Thus, in comparison with cultivated maize, its wild relative teosinte is a reservoir of genetic variation, and often exhibits favorable nutritional attributes [15], stress resilience [48, 49], and even agronomic and yield performance [12–14, 50]. Multiple favorable alleles from teosinte have been mined, such as *ZmWAK* for resistance to head smut [51], *ZmNAC111* for drought tolerance in maize seedlings [52], and *UPA2* for leaf angle [50]. Notably, the teosinte *UPA2* allele reducing the leaf angle, which has a pretty low allele frequency (4.4%) in teosinte that has not been used in modern maize, was introgressed into an elite modern maize hybrid, Nongda108, via marker-assisted selection, and finally enhanced the maize yield under dense planting [50]. It is a successful example to incorporate the teosinte germplasm to improve the maize breeding. These findings suggest the potential to identify other beneficial variants useful for maize genetic improvement that may be hidden in teosinte. The five species of teosinte in the genus *Zea*, *parviglumis*, *mexicana*, *huehuetenangensis*, *diploperennis* and *luxurians,* can be hybridized with modern maize [3], enabling the transfer of favorable alleles that currently exist in wild relatives into modern maize breeding pools.

Capitalizing on the development of efficient genotyping technology, teosinte represents an attractive system for the study of population and ecological genomics of maize domestication, introgressive hybridization, and local adaptation [3, 53]. In our study, different methods including ADMIXTURE analysis, PCA, and phylogenetic tree analysis clearly elucidated the genetic relationship between maize and its wild relatives based on over 40,000 SNPs across the genome. Consistent with previous studies [2, 5, 6, 31, 32], our results confirm a single domestication event in maize from the Central Balsas *parviglumis* race and favor the Balsas River valley as the center of maize domestication. Notably, the domestication process inferred from paleogenomic data was both gradual and complex, in which different genetic loci were selected at different time points, and the transformation of teosinte to maize was completed in the last 5000 years [54]. In addition to the evolutionary relationship between maize and teosinte, we also determined the evolutionary lineage of all species within teosinte, namely that *parviglumis* are closest to *mexicana*, followed in order by *huehuetenangsis*, *diploperennis* and *perennis*, *luxurians* and *nicaraguensis*. These findings answer a fundamental question in the taxonomic classification of teosintes, which has been debated during the last five decades [2, 55–59].

Our comparative genomic analysis between wild and modern maize, and between tropical and temperate maize, identified 5.7% of the genome that had been selected during maize domestication, and 5.5% of the genome that had been selected during adaptation. Our data cannot differentiate selective sweeps with domestication features from those with improvement features because we didn’t look at maize landraces. In comparisons to previous studies, the size of the selected genomic regions we identified is smaller, and only 24% (95/394) of putative domestication-related selective sweeps overlapped with the results of Hufford et al. [50], and 17% (62/360) of putative adaptation genes overlapped with the results of Liu et al. [23]. These low percentages may result from different genetic germplasms, sample sizes, and SNP densities as well as from differences in the quality of the reference genome (Table S3). Although the SNP density used was relatively low, the larger sample size in our study shows greater genetic diversity (Fig. S6) and could increase the power of detecting selection signals [60]. With newer developments in sequencing technology, re-sequencing our germplasm plus a set of maize landraces will refine what we are able to conclude about maize domestication, improvement, and adaptation.

Maize was subjected to drastic morphological or physiological changes during domestication that now differentiate it from its teosinte progenitor. Given these changes, the selective sweeps identified in this study could be associated with domestication and adaptation traits. These associations were supported by the co-location of the selective sweeps identified here and eight domestication genes (e.g., *tb1* [16–18] and *pbf1* [36]) plus a set of GWAS signals for flowering-time traits (Fig. 5). In addition to the known genes and existing GWAS signals reported in previous studies, ten candidate genes were identified that colocalized at both GWAS and selection signals. As an example, *GRMZM2G169293* had a genetic effect on flowering time that was dependent on altitude. Similar trends have been seen in known adaptation genes such as *ZmCCT10* [27] and *ZmCCT9* [28]. Such temperature-related highland adaptation loci could be important for maize breeding in the face of climate change [3]. Therefore, identification of selective sweeps during maize domestication and adaptation will extend our understanding of these processes, and greatly benefit maize breeding if this information is included in the process of maize improvement.

## Conclusions

In summary, we determined the genetic structure reflected the historical evolutionary relationships among *Zea* species and subspecies, namely that maize is closest to *parviglumis*, followed by *mexicana*, *huehuetenangsis*, *diploperennis* and *perennis*, *luxurians* and *nicaraguensis*. Our comparative population genomic studies identified more than 600 domestication and adaptation sweeps, and the existing GWAS hits for flowering time were highly enriched in the selective sweeps. Combining with the GWAS results, we identified ten candidate genes that were significantly associated with adaptation traits and that have undergone selection during maize adaptation. Notably, a candidate gene *GRMZM2G169293* was identified, which located within an adaptation selective sweep and was associated with photoperiod responses. Taken together, our results will provide increasing insights into the evolutionary history of maize and will greatly benefit the maize breeding.

## Materials and methods

### Plant material

A set of 982 maize lines and 190 teosinte accessions were used in this study. The maize lines, representative of tropical, subtropical, and temperate germplasm, were collected from maize breeding programs of the International Maize and Wheat Improvement Center (CIMMYT) (*n* = 691), China (*n* = 221), the USA (*n* = 66), Thailand (*n* = 3), and Peru (*n* = 1) (Data S4). The teosinte accessions, representative of the entire geographical distribution of teosinte across Mexico and Central America, included 12 *nicaraguensis*, one *luxurians*, three *diploperennis*, two *perennis*, one *huehuetenangsis*, 96 *mexicana*, and 75 *parviglumis* accessions (Data S4). Based on their geographical distribution, the *mexicana* accessions were further divided into five geographical groups from Puebla, Central Plateau, Chalco, Durango and Nobogame, and *parviglumis* accessions were also further divided into five geographical groups from Southern Guerrero, Oaxaca, Eastern Balsas, Central Balsas, and Jalisco (Fig. S1 and Data S4).

### Genotyping and SNP quality control

DNA was extracted from leaves that were obtained from a pool of at least six individuals for each maize line and one individual per teosinte accession. All maize lines and teosinte accessions were genotyped using the Illumina MaizeSNP50 BeadChip (Illumina Inc., San Diego, CA, USA) containing 56,110 SNPs [61]. SNP genotypes were manually checked as reported previously [62]. A total of 2353 SNPs with poor performance were removed from subsequent analyses. In addition, only the SNPs with probe sequences uniquely mapped to the B73 reference genome (B73 RefGenV3) using the Burrows-Wheeler Aligner (BWA) were retained [63]. A final set of 42,204 polymorphic and single-copy SNPs with < 20% missing data across all 1172 accessions was used in the final analyses. The PIC for each SNP was calculated using PowerMarker version 3.25 [64].

### Population structure analysis

Hierarchical population structure of all maize lines and teosinte accessions was estimated with the program ADMIXTURE, which implemented a Structure-like model-based maximum likelihood clustering algorithm [65]. The maize lines and teosinte accessions were subsequently analyzed separately. For maize, lines with membership probabilities ≥0.70 were assigned to that corresponding group, and lines with a probability of < 0.70 for both the temperate and tropical groups were assigned to a mixed group. For teosinte, the entries were assigned to the corresponding subspecies and geographical groups based on their known origins and ADMIXTURE results. ADMIXTURE results showing individual assignments to corresponding groups were graphically displayed using R Version 3.1.1 (www.R-project.org).

### Visualization of relationships

PCA was performed at the individual level using the GCTA software [66]. Subgroups were formed that included all maize and teosinte accessions, maize inbred lines only, teosinte accessions only, and teosinte accessions split into two subgroups. The first three principal components were used to visualize the genetic relatedness among individuals and to investigate the groups. The identity-by-state distance matrix was calculated between each pair of lines with PLINK Version 1.7 [67], and was then imported into the MEGA6 program [68] to construct a neighbor-joining phylogenetic tree. One *nicaraguensis* accession was used as the outgroup.

### Haplotype phasing and visualization

Haplotype phasing was done independently for each chromosome by SHAPEIT Version 2.12 [69, 70] with 2-Mb window size, 20 burn-in iterations, 20 iterations of the pruning stage, and 30 main iterations. Then the genome was divided into 50-kb windows to determine the haplotypes of linked SNPs in each window. If a window contains more than five SNPs, a random subset of five SNPs was selected for haplotype analysis, and the same randomly selected SNPs were used for all individuals. As a result, the SNP number used for haplotype analysis in each window ranged from one to five. For subsequent analyses, each haplotype window was defined as a locus, and each unique haplotype within the window was defined as an allele. In total, 17,109 loci were visualized for the window-based haplotypes.

### Genome scanning for regions that have undergone selection

To achieve maximum statistical power, XP-CLR hosted on GitHub [34] was implemented along with the population fixation statistic, *F*_ST_, using VCFtools [71] to detect loci that may have undergone selection during maize domestication and adaptation. In the analysis of XP-CLR, we used a 100-kb sliding window and a 10-kb step size. To ensure comparability of the composite likelihood score in each window, we fixed the number of SNPs assayed in each window to five with the setting ‘--maxsnps 5 --minsnps 5’ [34]. Meanwhile, to keep the used genomic windows consistent in the XP-CLR analysis, the weighted *F*_ST_ values were estimated in each window that required at least five SNPs with the setting ‘--fst-window-size 100,000 --fst-window-step 10,000’ [71]. Pairwise differentiation between populations (*F*_ST_) was calculated using the “hierfstat” package of R [72].

Evidence for selection across the genome during the domestication and adaptation processes were evaluated in two separate comparisons: teosinte versus maize for domestication and tropical maize lines versus temperate maize lines for adaptation. For each method, we merged the adjacent windows with top 10% values into a single window, and the top 0.5% outliers were determined to represent putative selection signals. In addition, adjacent sweeps separated by a physical distance of < 100 kb were merged into a single selected locus.

### Genome-wide association mapping for flowering-time traits

The 508 diverse inbred lines that made up an association mapping panel [73] were planted in seven environments, including six long-day (> 13 h) and one short-day (< 13 h) growing-season environments [27]. Flowering time was recorded as DTA and DTS, and these values were then converted into GDDs. APR and SPR were calculated as the difference between GDDs under long- and short-day conditions for pollen shed and silking, respectively. The best linear unbiased prediction (BLUP) values for each trait were used for the marker-trait association analysis. Using ~ 1.25 million previously reported SNPs with a minor allele frequency of ≥0.05 [45], the marker-trait association analyses were performed using a mixed linear model [74] presented in TASSEL 5.2 [75], which accounted for population structure and relative kinship [76]. Because the SNPs used for GWAS are in LD at different levels, we first performed LD pruning for the 1.25 million SNPs (window size 50, step size 50, *r*^2^ ≥ 0.2) using PLINK [67], and obtained 165,202 independent SNPs. Consequently, the Bonferroni-corrected threshold, 6.05 × 10^− 6^ (*P* < 1/165,202), was used as the whole-genome significance cutoff. Marker-trait associations were also analyzed with this dataset for flowering time in each environment.

### Data availability

SNP data for this study has been uploaded to European Variation Archive and can be retrieved through the project number PRJEB41335 (http://wwwdev.ebi.ac.uk/eva/?eva-study=PRJEB41335).

## Supplementary Information

**Additional file 1: Figure S1.** Geographical distribution of all teosinte accessions. **Figure S2.** Genetic relationships of maize and teosinte assessed by PCA. **Figure S3.** Evaluation of the ascertainment bias caused by Syngenta SNPs. **Figure S4.** Haplotype richness in maize and teosinte groups estimated via window-based methods. **Figure S5.** Co-localization of putative selective sweeps with public GWAS hits for flowering time. **Figure S6.** Genetic relationships of maize and teosinte assessed by PCA using 36,839 common SNPs between this study and Hufford et al.’s study. **Table S1.** Population divergence among maize and teosinte subgroups estimated by pairwise *F*_ST_ values between different groups. **Table S2.** List of known domestication, improvement and adaptation genes in maize. **Table S3.** Comparisons of selective sweeps identified in this study and previous studies, and the factors affecting the identification of selective sweeps.

**Additional file 2: Data S1.** Genetic relationship of maize and teosinte inferred by ADMIXTURE.

**Additional file 3: Data S2.** Summary of selection sweeps with domestication and adaptation features.

**Additional file 4: Data S3.** Summary of SNPs significantly associated with flowering-time traits detected by GWAS.

**Additional file 5: Data S4.** List of plant materials used in this study.

## Abbreviations

QTL: Quantitative trait locus
MITE: Miniature transposon
SNP: Single-nucleotide polymorphism
PCA: Principal component analysis
XP-CLR: Cross-population composite likelihood ratio
LD: Linkage disequilibrium
GWAS: Genome-wide association studies
NAM: Nested-association mapping
DTA: Days to anthesis
DTS: Days to silking
GDD: Growing degree days
APR: Anthesis photoperiod response
SPR: Silking photoperiod response
BLUP: Best linear unbiased prediction
MAF: Minor allele frequency
PIC: Polymorphic information content
