# Supplementary material for: Population genomics of Zea species identifies selection signatures during maize domestication and adaptation
Source: BMC Plant Biol. 2022 Feb 18;22:72. doi: 10.1186/s12870-022-03427-w (PMC8855575; doi:10.1186/s12870-022-03427-w)
Supplement: Supplementary file 1 — Additional file 1: Figure S1. Geographical distribution of all teosinte accessions. Figure S2. Genetic relationships of maize and teosinte assessed by PCA. Figure S3. Evaluation of the ascertainment bias caused by Syngenta SNPs. Figure S4. Haplotype richness in maize and teosinte groups estimated via window-based methods. Figure S5. Co-localization of putative selective sweeps with public GWAS hits for flowering time. Figure S6. Genetic relationships of maize and teosinte assessed by PCA using 36,839 common SNPs between this study and Hufford et al.’s study. Table S1. Population divergence among maize and teosinte subgroups estimated by pairwise FST values between different groups. Table S2. List of known domestication, improvement and adaptation genes in maize. Table S3. Comparisons of selective sweeps identified in this study and previous studies, and the factors affecting the identification of selective sweeps. [file 12870_2022_3427_MOESM1_ESM.docx]

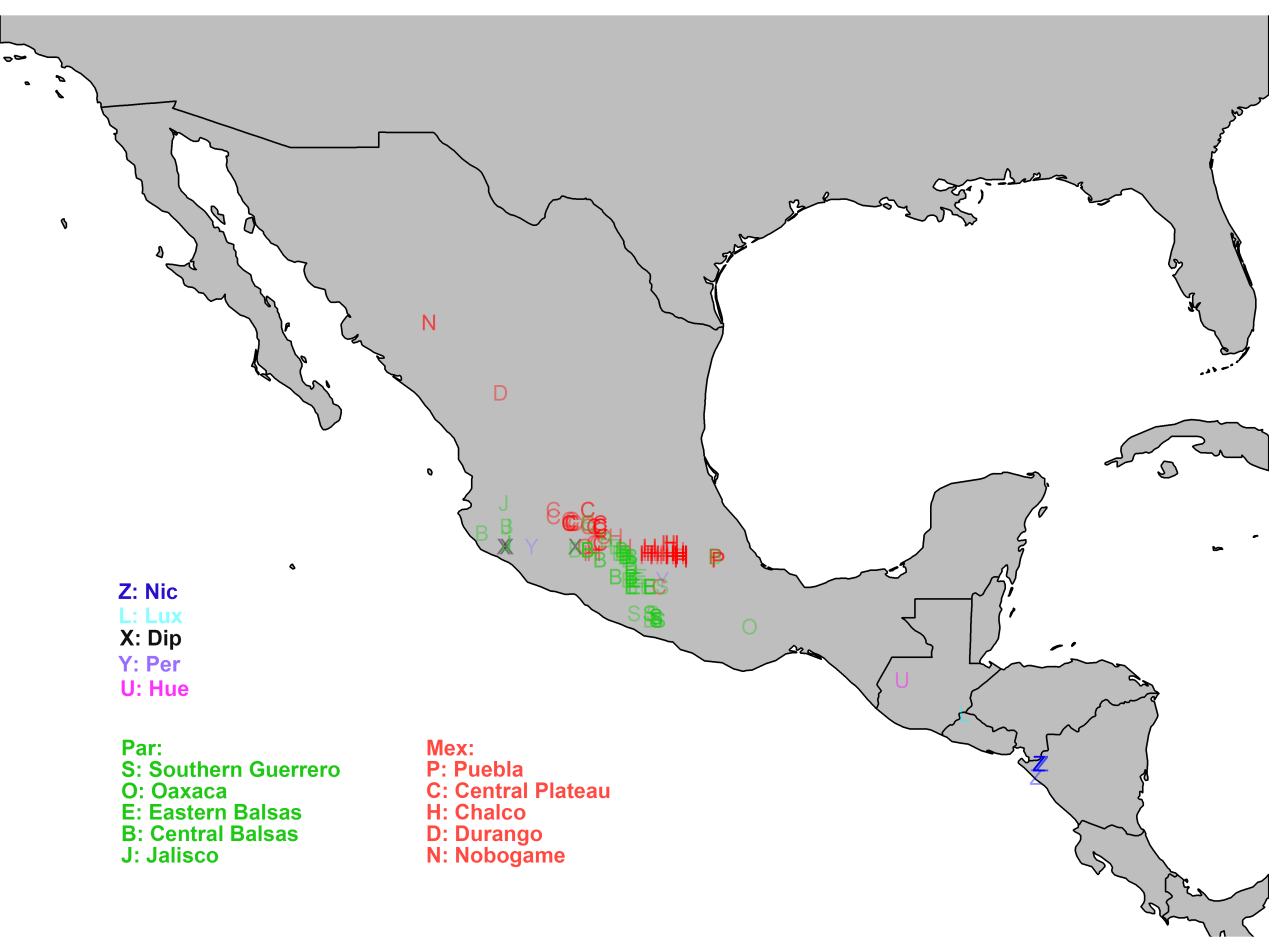


**Figure S1 Geographical distribution of all teosinte accessions.** Nic, *nicaraguensis*; Lux, *luxurians*; Dip, *diploperennis*; Per, *perennis*; Hue, *huehuetenangsis*; Mex, *mexicana*; Par, *parviglumis*. The *parviglumis* and *mexicana* races are defined in the key: Par_S, Southern Guerrero; Par_E, Eastern Balsas; Par_O, Oaxaca; Par_B, Central Balsas; Par_J, Jalisco; Mex_P, Puebla; Mex_C, Central Plateau; Mex_D, Durango; Mex_H, Chalco; Mex_N, Nobogame. The detailed georgraphical information for teosinte is listed in **Data S4**.


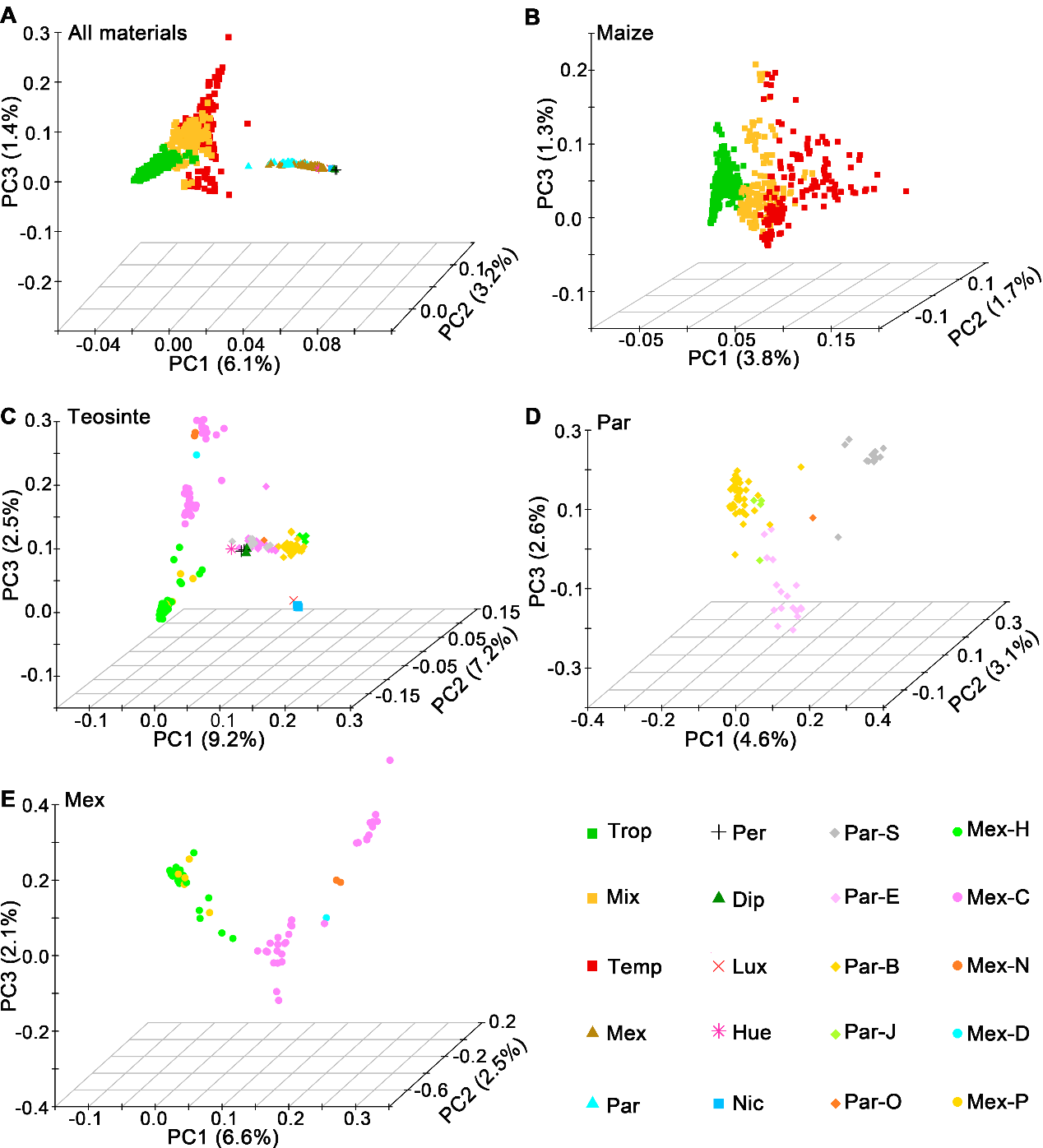


**Figure S2** **Genetic relationships of maize and teosinte assessed by PCA**. **(A)** A PCA plot for all entries (982 maize lines and 190 teosinte accessions). **(B−E)** PCA plots for maize **(B)**, teosinte **(C)**, *parviglumis* **(D)** and *mexicana* **(E)**. All entries are marked according to the inferred clusters from the ADMIXTURE analysis. Trop, tropical maize; Temp, temperate maize; Mix, maize lines with membership probabilities in both tropical and temperate groups of <0.70; Nic, *nicaraguensis*; Lux, *luxurians*; Dip, *diploperennis*; Per, *perennis*; Hue, *huehuetenangsis*; Mex, *mexicana*; Par, *parviglumis*. The *parviglumis* races: Par_S, Southern Guerrero; Par_E, Eastern Balsas; Par_O, Oaxaca; Par_B, Central Balsas; Par_J, Jalisco. The *mexicana* races: Mex_P, Puebla; Mex_C, Central Plateau; Mex_D, Durango; Mex_H, Chalco; Mex_N, Nobogame.


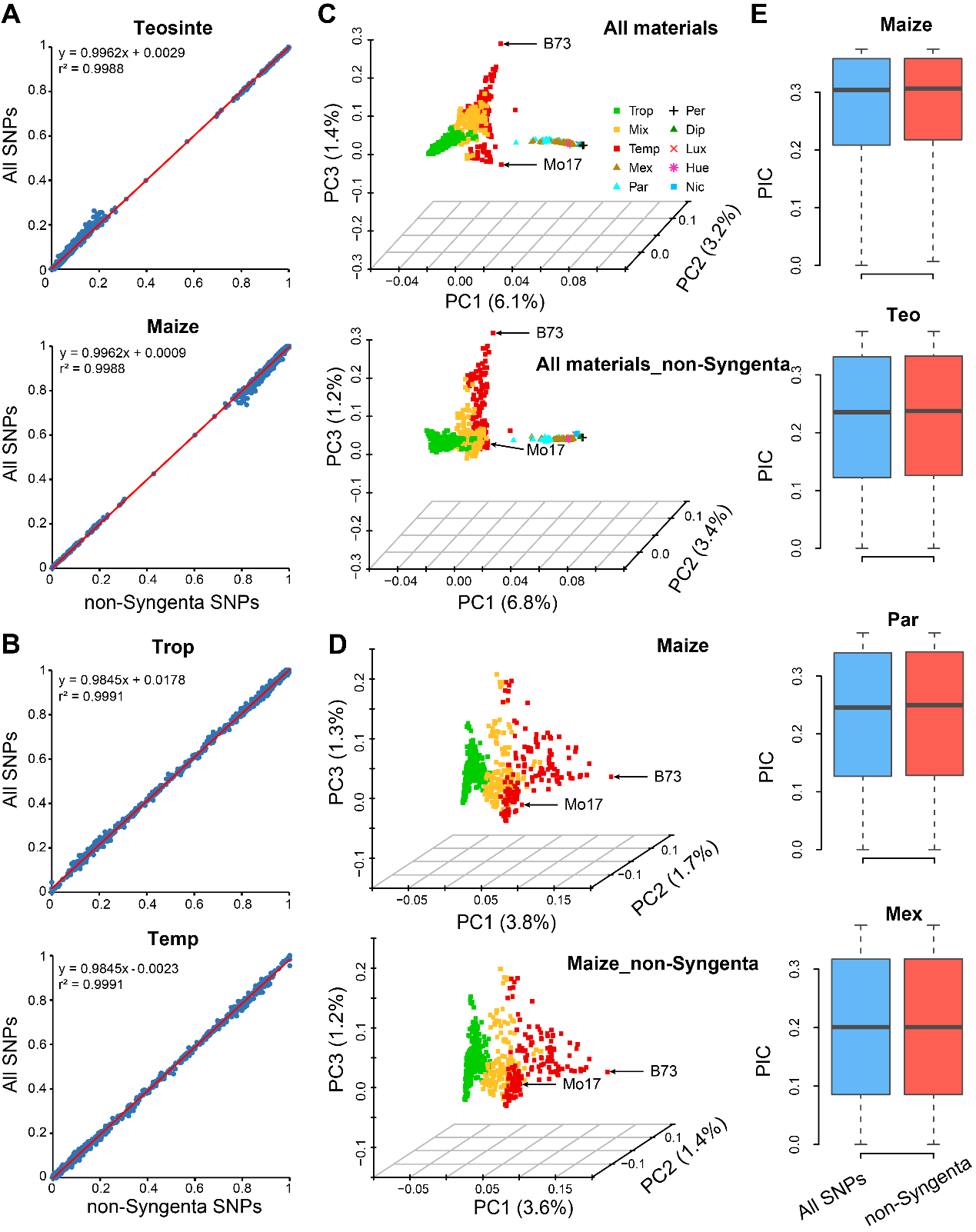


**Figure S3 Evaluation of the ascertainment bias caused by Syngenta SNPs.** (**A-B**) Correlation analysis of membership probabilities calculated by all SNPs and non-Syngenta SNPs in each assigned group. (**C-D**) PCA plots for all materials (**C**) and maize inbred lines (**D**) assessed using all SNPs (up panel) and non-Syngenta SNPs (down panel). (**E**) Distribution of polymorphic information content for all SNPs and non-Syngenta SNPs in each population. Trop, tropical maize; Temp, temperate maize; Par, *parviglumis*; Mex, *mexicana*.


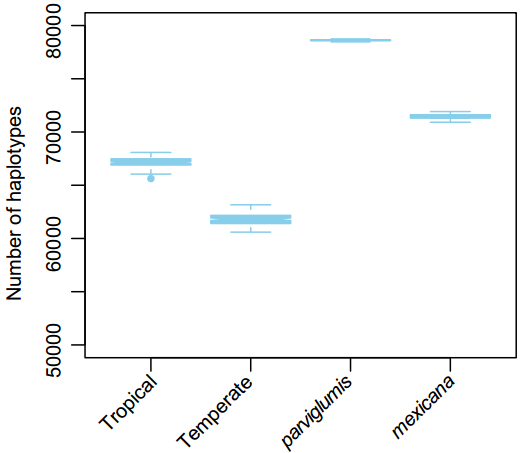


**Figure S4** **Haplotype richness in maize and teosinte groups estimated via the window-based method.** A total of 75 samples were randomly selected from tropical maize, temperate maize, and *mexicana* accessions with 100 bootstraps.


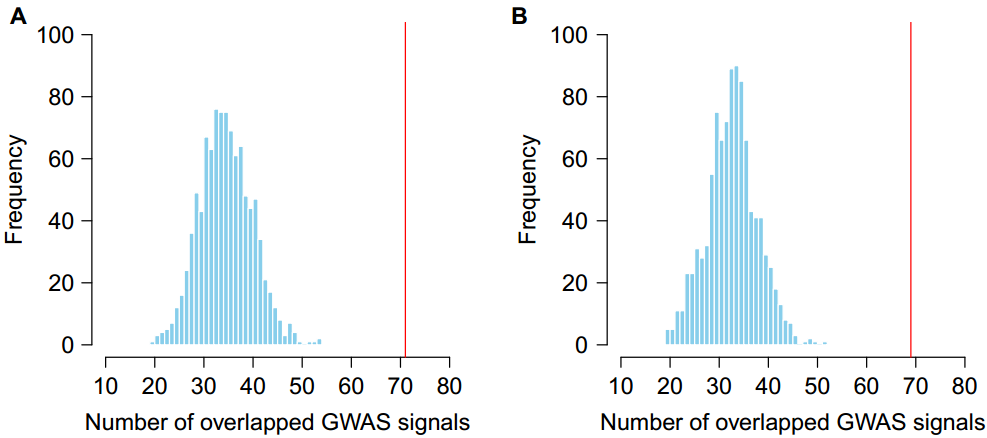


**Figure S5** **Co-localization of putative selective sweeps with public GWAS hits for flowering time.** Counts of the public GWAS hits for flowering time [27, 39, 40] in the domestication (**A**) and adaptation (**B**) sweeps (red vertical lines) were compared with 1,000 permutations of controls (blue histogram).

**
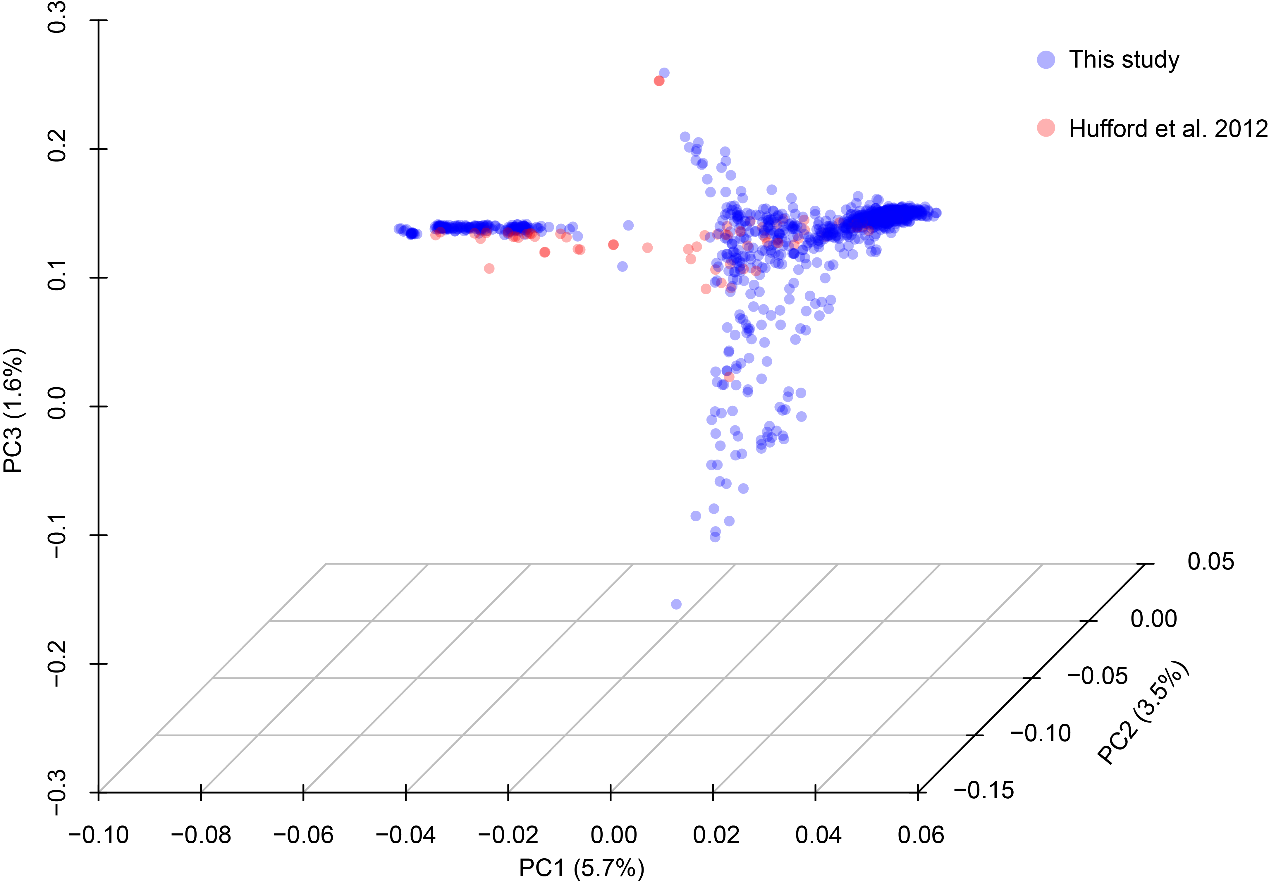
**

**Figure S6 Genetic relationships of maize and teosinte assessed by PCA using 36,839 common SNPs between this study and Hufford et al.’s study [47].** The blue dots show the samples in this study, while the red dots show the samples in Hufford et al.’s study [47]. The PCA plot shows that the germplasms used in this study harbor greater genetic diversity than that in Hufford et al.’s study [47].

**Table S1** **Population divergence among maize and teosinte subgroups estimated by pairwise *F*_ST_ values between different groups**

| Subgroup | *mexicana* | *parviglumis* | Tropical maize |
| --- | --- | --- | --- |
| *parviglumis* | 0.10 |  |  |
| Tropical maize | 0.19 | 0.17 |  |
| Temperate maize | 0.21 | 0.19 | 0.12 |

**Table S2 List of known domestication and adaptation genes in maize**

| **Gene name** | **Gene ID_V3** | **Chr** | **Pos.start.V3** | **Pos.end.V3** | **Selection Features** | **Reference** |
| --- | --- | --- | --- | --- | --- | --- |
| *zagl1* | *GRMZM2G026223* | 1 | 4855703 | 4871281 | Domestication | Zhao *et al.*, 2011, *Genet. Res.*, 93, 65-75; Wills *et al.*, 2018, *J. Hered.*, 109, 333-338 |
| *gt1* | *GRMZM2G005624* | 1 | 23240104 | 23243489 | Domestication | Whipple *et al.*, 2011, *Proc. Natl. Acad. Sci. USA*, 108, E506-512; Wills *et al.*, 2013, *PLoS Genet.*, 9, e1003604 |
| *zmm6* | *GRMZM2G159397* | 1 | 194046502 | 194054589 | Domestication | Zhao et al., 2011, *Genet. Res.*, 93, 65-75 |
| *ZmSh1-1* | *GRMZM2G085873* | 1 | 260424101 | 260427079 | Domestication | Lin *et al.*, 2012, *Nat. Genet.*, 44, 720-724; Yang *et al.*, 2016, *Genetics*, 204, 1573-1585 |
| *tb1* | *AC233950.1_FG002* | 1 | 265811311 | 265813044 | Domestication | Studer *et al.*, 2011, *Nat. Genet.*, 43, 1160-1163 |
| *D8* | *GRMZM2G144744* | 1 | 266160101 | 266163168 | Adaptation | Camus-Kulandaivelu *et al.*, 2008, *Genetics*, 180, 1107-1121 |
| *ids1/Ts6* | *GRMZM5G862109* | 1 | 292966682 | 292970925 | Domestication | Wang *et al.*, 2019, *New Phytol.*, 223, 1634-1646 |
| *pbf1* | *GRMZM2G146283* | 2 | 154144472 | 154148029 | Domestication | Jaenicke-Després *et al.*, 2003, *Science*, 302, 1206-1208 |
| *zag2* | *GRMZM2G160687* | 3 | 137255997 | 137261748 | Domestication | Zhao et al., 2011, *Genet. Res.*, 93, 65-75 |
| *tru1* | *GRMZM2G039867* | 3 | 150088574 | 150091550 | Domestication | Dong *et al.*, 2017, *Proc. Natl. Acad. Sci. USA*, 114, E8656-E8664 |
| *ZmMADS69* | *GRMZM2G171650* | 3 | 159022119 | 159050063 | Domestication | Zhao *et al.*, 2011, *Genet. Res.*, 93, 65-75; Liang *et al.*, 2019, *New Phytol.*, 221, 2335-2347 |
| *su1* | *GRMZM2G138060* | 4 | 41396390 | 41405179 | Domestication | Whitt *et al.*, 2002, *Proc. Natl. Acad. Sci. USA*, 99, 12959-12962; Jaenicke-Després *et al.*, 2003, *Science*, 302, 1206-1208 |
| *tga1* | *GRMZM2G101511* | 4 | 44534815 | 44539478 | Domestication | Wang *et al.*, 2005, *Nature*, 436, 714-719 |
| *bt2* | *GRMZM2G068506* | 4 | 58979526 | 58985686 | Domestication | Whitt *et al.*, 2002, *Proc. Natl. Acad. Sci. USA*, 99, 12959-12962 |
| *zmm19* | *GRMZM2G370777* | 4 | 178918189 | 178925234 | Domestication | Zhao et al., 2011, *Genet. Res.*, 93, 65-75 |
| *KRN4* | *GRMZM2G460544* | 4 | 199456664 | 199460958 | Domestication | Liu *et al.*, 2015, *PLoS Genet.*, 11, e1005670 |
| *ZmSWEET4c* | *GRMZM2G137954* | 5 | 127500983 | 127504476 | Domestication | Sosso *et al.*, 2015, *Nat. Genet.*, 47, 1489-1493 |
| *ae1* | *GRMZM2G032628* | 5 | 168492139 | 168509225 | Domestication | Whitt *et al.*, 2002, *Proc. Natl. Acad. Sci. USA*, 99, 12959-12962 |
| *ZmMADS2* | *GRMZM2G316366* | 5 | 177268178 | 177269968 | Domestication | Zhao et al., 2011, *Genet. Res.*, 93, 65-75 |
| *zag1* | *GRMZM2G052890* | 6 | 132008587 | 132016861 | Domestication | Zhao *et al.*, 2011, *Genet. Res.*, 93, 65-75 |
| *DXS2* | *GRMZM2G493395* | 7 | 14086686 | 14089909 | Domestication | Fang *et al.*, 2020, *Plant J.*, 101, 278-292 |
| *ra1* | *GRMZM2G003927* | 7 | 110359182 | 110359930 | Domestication | Sigmon and Vollbrecht, 2010, *Mol. Ecol.*, 19, 1296-1311 |
| *zmm28* | *GRMZM2G147716* | 7 | 164451097 | 164456819 | Domestication | Zhao et al., 2011, *Genet. Res.*, 93, 65-75 |
| *ZCN8* | *GRMZM2G179264* | 8 | 123030387 | 123032175 | Domestication and Adaptation | Guo *et al*., 2018, *Curr. Biol.*, 28, 3005-3015 e3004 |
| *Vgt1/ ZmRap 2.7* | *GRMZM2G700665* | 8 | 131576889 | 131580316 | Adaptation | Guo *et al*., 2018, *Curr. Biol.*, 28, 3005-3015 e3004; Salvi *et al*., 2017, *Proc. Natl. Acad. Sci. USA*, 104, 11376-11381 |
| *zmm3* | *AC197699.3_FG001* | 9 | 17021226 | 17024054 | Domestication | Zhao et al., 2011, *Genet. Res.*, 93, 65-75 |
| *GL15* | *GRMZM2G160730* | 9 | 96744684 | 96748035 | Domestication | Xu *et al.*, 2017, *Mol. Plant*, 10, 1579-1583 |
| *ZmCCT9* | *GRMZM2G004483* | 9 | 115786897 | 115789787 | Adaptation | Huang *et al.*, 2018, *Proc. Natl. Acad. Sci. USA*, 115, E334-E341 |
| *ZmCCT10* | *GRMZM2G381691* | 10 | 94262291 | 94264845 | Adaptation | Yang *et al.*, 2013, *Proc. Natl. Acad. Sci. USA*, 110, 16969-16974 |

**Table S3 Comparisons of selective sweeps identified in this study and previous studies, and the factors affecting the identification of selective sweeps**

| Studies | Approaches for selection scan | Sample size and used germplasms | SNP density | Reference genome | Sweep number | |
| --- | --- | --- | --- | --- | --- | --- |
|  |  |  |  |  | Domestication | Adaptation |
| This study | XP-CLR/ *F*_ST_ | 982 maize inbred lines, 171 teosinte accessions | 42,204 | Version 3 | 394 | 360 |
| Hufford et al. 2012 [47] | XP-CLR | 35 improved maize lines, 23 landraces, 14 wild relatives | 21,141,953 | Version 2 | 484 | - |
| Liu et al. 2015 [45] | XP-CLR | 368 maize inbred lines | 558,529 | Version 2 | - | 695 |
